# Supplementary material for: Patterns of Intron Gain and Loss in Fungi
Source: PLoS Biol. 2004 Nov 30;2(12):e422. doi: 10.1371/journal.pbio.0020422 (PMC532390; doi:10.1371/journal.pbio.0020422)
Supplement: Table S1 — Also available at http://genes.mit.edu/NielsenEtAl/. (4.3 MB ZIP). [file pbio.0020422.st001.zip › NielsenEtAl/html/627.html]

AN3134.1.NCU01843.1.MG06639.1.FG05985.1


```
 CLUSTAL W (1.82) Multiple Sequence Alignments - Introns Inserted


Sequence 1: NCU01843.1	543 aa
Sequence 2: MG06639.1	541 aa
Sequence 3: FG05985.1	538 aa
Sequence 4: AN3134.1	538 aa
Alignment Length: 544 aa
Number Identitical Residues: 409 aa
Alignment Score (without introns) 17200


MG06639.1 	MQAPVVVMN1TQSGGERQTGRKAQLSNIAAAKI2VADIIRSCLGPKAMLKMLLDPMGGIV
NCU01843.1	MQAPVLVMN1TNSG-ERQTGRKAQMSNIAAAKT2VADIIRSCLGPKAMLKMLLDPMGGIV
FG05985.1 	MQAPVVVMN1TNSG-ERQTGRKAQLSNIAAAKT2VADIIRSCLGPKAMLKMLLDPMGGIV
AN3134.1  	MQAPVVVMN1TNSG-ERQVGRKAQLSNITAAKT2VADIIRSCLGPKAMLKMLLDPMGGIV
          	*****:*** *:** ***.*****:***:***  **************************

MG06639.1 	LTNDGHAILREIEVSHPAAKSMIELSRTQDEE~VGDGTTS---VIILG~GEILAQALPQL
NCU01843.1	LTNDGHAILREIEVSHPAAKSMIELSRTQDEE0LRRIPFTDRWYSQTA~GEILAQSLPQL
FG05985.1 	LTNDGHAILREIEVSHPAAKSMIELSRTQDEE~VGDGTTT---VIILA1GEILAQALPQL
AN3134.1  	LTNDGHAILREIEVSHPAAKSMIELSRTQDEE~VGDGTTT---VIILA1GEMLAQALPQL
          	******************************** :   . :       . **:***:****

MG06639.1 	ERNIHPVVIISAFKRALKDALEIIDEISTPIDINDDEAMYQLISSSIGTKFVSRWSKLMC
NCU01843.1	ERNIHPVVIISAFKRALKDALQIIEDISLPIDINDDQAMYKLISSSIGTKYVSRWSELMC
FG05985.1 	DRNIHPVVIISAFKRALKDALEIIEEISLPIDVNDDKAMYQLISSSIGTKFVSRWMDQMC
AN3134.1  	ERNIHPVVIISAFKRALADALAIIEEVSLPVDIDDDKAMYTLIKSSIGTKFVSRWSDLMC
          	:**************** *** **:::* *:*::**:*** **.******:**** . **

MG06639.1 	SLALKAVRTVTWEAGTGKREVDIKRYARIEKVPGGEIEDSRVLDGVMLNKDITHPKMRRR
NCU01843.1	GLALKAVRTVTWEQGNGKKEVDIKRYARVEKVPGGEIEDSRVLDGVMLNKDITHPKMRRR
FG05985.1 	DLALKAVRTVTWEAGNGKTEVDIKRYARVEKVPGGEIEDSRVLDGLMLNKDITHPKMRRR
AN3134.1  	DLALKAVRTVSFDAGGGKREVDIKRYARIEKIPGGQIEDSEVIDGVMVNKDITHPKMRRR
          	.*********::: * ** *********:**:***:****.*:**:*:************

MG06639.1 	IENPRIILLDCPLEYKKGESQTQIEITKEEDWNRILQIEEEQVEAMCKHILELKPDLVIT
NCU01843.1	IENPRIILLDCPLEYKKGESQTNIEITKEEDWNRILEIEEEQVKQMCEHILAFNPDLVIT
FG05985.1 	IENPRIVLLDCPLEYKKGESQTNIEITKEDDWNRILQIEEEQVKALCEAIVAVKPDLVIT
AN3134.1  	IENPRVILLDCPLEYKKGESQTNIEITKEDDWNRILQIEEEQVKHMCDAILALKPDVVIT
          	*****::***************:******:******:******: :*. *: .:**:***

MG06639.1 	EKGVSD~LAQHYFMKANVTALRRVRKTDNNRIARATGATIVNRVEDLQESDVGTQCGLFE
NCU01843.1	EKGVSD1LAQHYLMKANVTALRRVRKTDNNRIARATGATIVNRVEDLQESDVGTNCGLFE
FG05985.1 	EKGVSD1LAQHYFMKANITALRRVRKTDNNRIARATGATIVNRVEDLQDSDVGTRCGLFE
AN3134.1  	EKGVSD1LAQHFLMKANVTAIRRVRKTDNNRIARATGATIVNRVDDIQESDVGTGCGLFE
          	****** ****::****:**:***********************:*:*:***** *****

MG06639.1 	IEKIGDEYFTFLTKCNTPKACTVLLRGPSKDILNEVERNLQDAMGVARNVMFHPRLSPGG
NCU01843.1	VEKIGDEYFTFLTKCKDPKACTVLLRGPSKDVLNEIERNLQDAMGVARNVMFHPRLSPGG
FG05985.1 	IEKIGDEYFTFLTKCQDPKACTVLLRGPSKDVLNEIERNLQDAMGVARNVMFSPKLSPGG
AN3134.1  	IEKIGDEYFTFLRKCQNPKACTILLRGPSKDIINEIERNLQDAMSVARNVIFHPRLCPGG
          	:*********** **: *****:********::**:********.*****:* *:*.***

MG06639.1 	GATEMAVSVRLGQLAKSIEGVQQWPYRAVAEALEVIPRTLVQNAGASPVRVLTDLRAKHA
NCU01843.1	GATEMAVSVRLTQLAKSIEGVQQWPYKAVAEALEVIPRTLVQNAGKSPVRVLTDLRAKHA
FG05985.1 	GATEMAVSVRLGQLAKSIEGVQQWPYKAVADALEVIPRTLVQNAGKSPVRVLTDLRAKQA
AN3134.1  	GAIEMAVSVKLGQLAKSIEGVQQWPYKAVADAMEIIPRTLAQNAGASPIRVLTRMRAKHV
          	** ******:* **************:***:*:*:*****.**** **:**** :***:.

MG06639.1 	EGKSSWGINGDTGGLADMKEYGVWEPEAIKVQSMKTAIE0AACLLLRVDDICSAKKAQPG
NCU01843.1	EGKNSWGINGDTGAIVDMKDYGVWEPEAIKVQSMKTAIE0AACLLLRVDDICSARKAQPG
FG05985.1 	EGKTTYGVNGDTGAVADMKDYGVWEPQAIKLQSIKTAIE0AACLLLRVDDICSAKKAAQ-
AN3134.1  	EGHTTWGLDGDTGALVDMKEYGVWEPEAVKLQSIKTAVE0SACLLLRVDDICSGKTAQQ-
          	**:.::*::****.:.***:******:*:*:**:***:* :************.:.*   

MG06639.1 	VGTGMTGGDD
NCU01843.1	VGVSSGGGDE
FG05985.1 	IGGG-GGGEE
AN3134.1  	-AAAPTGGDD
          	 . .  **::
```
